# Circadian rhythm of salivary cortisol, plasma cortisol, and plasma ACTH in end-stage renal disease

**DOI:** 10.1530/EC-12-0058

**Published:** 2012-11-19

**Authors:** Hershel Raff, Hariprasad Trivedi

**Affiliations:** 1 Division of Endocrinology, Department of Medicine Medical College of Wisconsin Milwaukee, Wisconsin, 53226 USA; 2 Department of Surgery Medical College of Wisconsin Milwaukee, Wisconsin, 53226 USA; 3 Department of Physiology Medical College of Wisconsin Milwaukee, Wisconsin, 53226 USA; 4 Division of Nephrology, Department of Medicine Medical College of Wisconsin Milwaukee, Wisconsin, 53226 USA; 5 Endocrine Research Laboratory Aurora St Luke's Medical Center 2801 W Kinnickinnic Parkway, Suite 245, Milwaukee, Wisconsin, 53215USA

**Keywords:** cortisol, circadian rhythm, ACTH, C-reactive protein, end-stage renal disease

## Abstract

**Objective:**

Patients with end-stage renal disease (ESRD) can display the features of endogenous hypercortisolism but are difficult to evaluate for Cushing's syndrome. We evaluated the circadian rhythm of plasma compared with salivary cortisol in subjects with ESRD.

**Design:**

Plasma and salivary cortisol and plasma ACTH samples were drawn frequently over 24 h in an inpatient research unit in stable ESRD subjects on daytime chronic hemodialysis (*n*=16) vs controls (*n*=8).

**Methods:**

Plasma cortisol was measured every 2 h from 0800 to 0600 h the following day. Salivary cortisol was measured every 2 h, except between 2400 and 0400 h (sleep time). Plasma ACTH measured in a subset of samples and C-reactive protein (CRP) was measured as a marker of a subclinical inflammatory state in all subjects.

**Results:**

ESRD subjects had a discernable circadian rhythm in plasma and salivary cortisol, but with a significantly higher nadir (1800–2400 h) compared with the controls (*P*=0.016–<0.001). After excluding four ESRD subjects without a normal circadian rhythm, the ESRD subjects still had higher nadir plasma and salivary cortisol and plasma ACTH compared with controls. There was no difference in the correlation of salivary and plasma cortisol in control vs ESRD subjects. ESRD subjects had higher CRP levels compared with controls.

**Conclusions:**

ESRD subjects had increased late-night plasma and salivary cortisol and plasma ACTH levels. Late-night salivary cortisol is a reliable index of plasma cortisol in ESRD patients.

## Introduction

The measurement of salivary cortisol has emerged as a first-line test in the evaluation of the hypothalamic–pituitary–adrenal (HPA) axis in humans and, in particular, for the diagnosis of endogenous hypercortisolism (Cushing's syndrome) (1, 2, 3, 4, 5, 6, 7). It is now accepted that an increased late-night salivary cortisol (at the circadian nadir) has 90–95% sensitivity and specificity for the diagnosis of Cushing's syndrome (6, 7).

In patients with normal renal function, salivary cortisol concentration is highly correlated with plasma free cortisol (7). However, its value in the assessment of possible endogenous hypercortisolism in patients with end-stage renal disease (ESRD) is not known. Other tests of HPA axis function are problematic in patients with diminished renal function. The inability to assess 24-h urine free cortisol in an anuric patient is self-evident. Furthermore, assessment of serum cortisol after dexamethasone suppression is also difficult to validate as plasma binding protein concentrations and dexamethasone clearance can be significantly altered with decreased renal function (8, 9, 10). Our reference laboratory often receives inquiries as to whether late-night salivary cortisol can be used to evaluate the possibility of endogenous Cushing's syndrome in patients with chronic kidney failure and we have been unable to provide a definitive answer based on previous studies (1, 2).

In patients with ESRD on chronic dialysis therapy, the mortality rate has remained close to 20% per year, about half due to cardiovascular diseases (11, 12, 13). In addition, uremic morbidities are extensive and include hypertension, metabolic bone disease, anemia, immunosuppression, and neuromuscular, dermatological, and endocrine disturbances (12, 13). Although the causes of morbidity and mortality are multifactorial, the types of morbidities suggest that increased cortisol secretion could play a contributory role. In fact, it has been observed that there are significant similarities between the morbidity of Cushing's syndrome and renal failure (8, 9). Thus, it is important to be able to properly assess the HPA axis in ESRD subjects.

The current study was designed to evaluate the circadian rhythm of the HPA axis using an intensive blood sampling protocol over 22 h in subjects with ESRD compared with a group of control subjects with normal renal function. Simultaneous salivary and plasma cortisol samples were taken when the subjects were awake to determine the value of salivary cortisol in the assessment of HPA axis in patients with ESRD.

## Materials and methods

### Subjects

The study was approved by the Medical College of Wisconsin Institutional Review Board and informed written consent was obtained from all subjects. Subjects were recruited from patients during usual clinical care or by referral from colleagues. Controls were recruited by referral from colleagues, volunteering employees, and from volunteers who heard about the study by word of mouth. The study population consisted of adult (age 18 years and older) subjects on chronic maintenance hemodialysis therapy for at least 30 days (*n*=16) and control subjects (*n*=8) without a history of kidney disease with an estimated glomerular filtration rate >70 ml/min per 1.73 m^2^. The primary outcome of the study was the comparison of the circadian rhythm of plasma and salivary cortisol. Exclusions included diabetes mellitus, subjects on oral corticosteroid preparations, pregnant women (serum pregnancy testing performed in women of child-bearing age), or unwillingness to participate and adhere to the protocol requirements. Subjects were not known to use inhaled steroids. Topical ointments and creams were not used during the sampling protocol. For 5 days before admission to the adult Translational Research Unit (TRU), subjects consumed a research diet tailored to contain 1000 mg phosphorus and 1200 mg calcium per day. In all other respects, the diet was similar to their usual diet.

### Study day

Subjects were admitted to the adult TRU the evening before the study day. ESRD subjects were sampled on a non-dialysis day; this was usually the day after their normal dialysis day. In the morning (∼0700 h) after TRU admission, an intravenous catheter was placed. Blood was sampled for plasma cortisol every 2 h from 0800 to 0600 h the following morning. Additional testing for plasma ACTH was done from blood drawn at 0800, 1200, 1800, 2200, 2400, and 0600 h. Salivary samples for cortisol were obtained using the Salivette (Sarstedt, Nümbrecht, Germany) every 2 h except for the 2400–0400 h samples while the subjects were sleeping. Breakfast was provided at ∼0800 h, lunch was provided at ∼1200 h (after the blood draw), dinner was provided at ∼1700 h, and a late evening snack was provided at ∼2000 h. A sleep pattern history was noted by the TRU nursing staff.

### Study day hormone measurements

Salivary cortisol and plasma ACTH were measured by validated immunoassays described in detail previously (14, 15). Plasma cortisol was measured using a Corti-cote assay (MP Biomedical, Solon, OH, USA). The sensitivity of this assay is 1.9 nmol/l, intra-assay precision 4.7–7.9%, and interassay precision 6.6–7.6%. Cross-reactivity was cortisol 100%, prednisolone 94.1%, prednisone 1.2%, 11-deoxycortisol 2.2%, prednisone 1.2%, and corticosterone 1.2%; all other cross-reactivities were <1.0%. Plasma human C-reactive protein (CRP) was measured by enzyme immunoassay (Quantikine, R&D Systems, Minneapolis, MN, USA). The minimum detectable concentration is 0.1 nmol/l, intra-assay precision 3.8–8.3%, and interassay precision 6.0–7.0%. There are no known significant cross-reactants. The reference range is reported to be 1.0–40.0 nmol/l.

### Calculations and statistical analysis

Criteria for a normal circadian rhythm were based on previous studies (7, 14, 16) as follows: plasma cortisol at 2400 h<210 nmol/l, salivary cortisol at 2200 h<6.0 nmol/l, ratio of 0800:2200 h plasma, and/or salivary cortisol >2. To be as conservative as possible and since salivary cortisol results were considered the dependent variable in the comparison of salivary and plasma cortisol, the upper cutoff for late-night salivary cortisol was set at the 99% confidence limit rather than 97.5% confidence limit (<4.2 nmol/l) used for the clinical reference range (14). Baseline data were compared by Mann–Whitney rank sum test and Kruskal–Wallis one-way ANOVA on ranks (Sigmaplot 11.0; Systat Software, Inc., San Jose, CA, USA). Circadian rhythm data were evaluated by two-factor ANOVA repeated on one factor (time) followed by pairwise multiple comparison using the Holm–Sidak method; data not normally distributed were evaluated after logarithmic transformation. Rates and proportions were analyzed by *χ* ^2^ or Fisher's exact test. Linear regression/correlations were analyzed by Spearman's rank-order correlation and, where appropriate, with regression slopes and *y*-intercepts compared by *t*-test. *P*<0.05 was considered significant. Graphical data are presented as mean/s.e.m. whereas tabular data are presented as median (25–75% confidence intervals).

## Results

Subject characteristics and baseline study day data are shown in Table 1. The ESRD subjects and controls were well matched in terms of the primary matching criteria (demographic characteristics), although the ESRD subjects tended to be younger (*t*-test, *P*=0.076; rank sum test, *P*=0.046). The causes of ESRD included focal segmental glomerulosclerosis (*n*=5), hypertension (*n*=6), chronic interstitial nephritis (*n*=1), chronic glomerulonephritis (*n*=1), polycystic kidney disease (*n*=1), post-streptococcal glomerulonephritis (*n*=1), and unknown (*n*=1).

**Figure 1:**
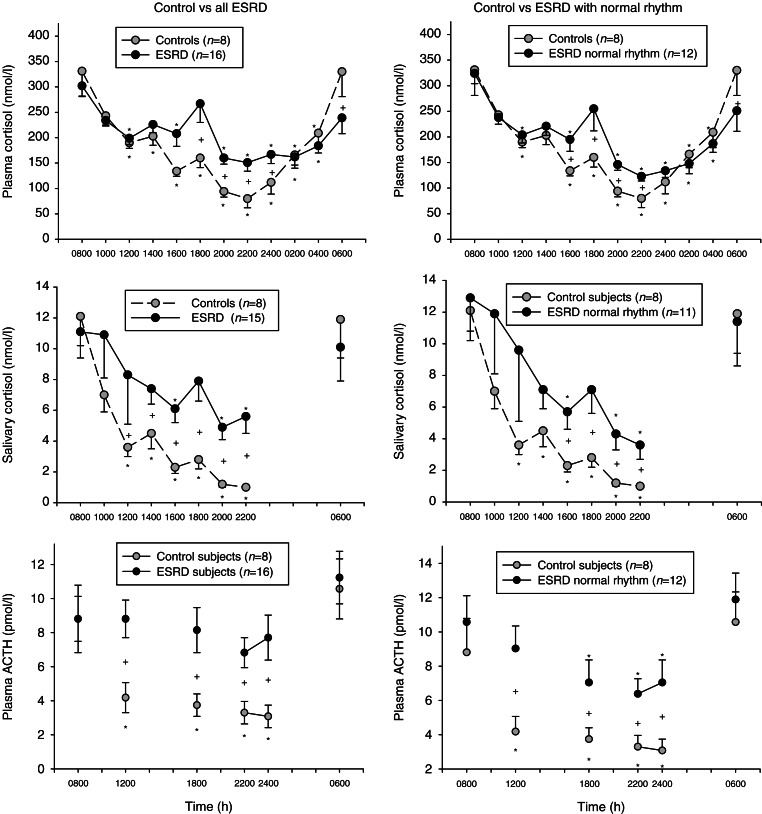
Plasma cortisol (top), salivary cortisol (middle), and plasma ACTH (bottom) in control subjects (*n*=8) compared to all ESRD subjects (left; *n*=16) or the subset of ESRD subjects who met the criteria for normal cortisol circadian rhythm (right; *n*=12). *Difference from 0800 h and ^+^difference between control and ESRD subjects at the same time point (*P*<0.05). Note that salivary cortisol was not sampled between 2400 and 0400 h (sleep time) and plasma ACTH was not assessed at all time points.

Figure 1 (left panels) shows the plasma and salivary cortisol and plasma ACTH results in control and ESRD subjects sampled every 2 h from 0800 h on day 1 to 0600 h on day 2. Note that salivary cortisol was not sampled from 2400 to 0400 h when the subjects were sleeping. Also note that one ESRD subject was not included in the salivary cortisol analysis because of obvious contamination of the salivary cortisol samples as described previously (17). The control subjects had typical circadian plasma and salivary cortisol and plasma ACTH rhythm patterns. The patterns of plasma compared to salivary cortisol were similar. The ESRD subjects also had a discernable circadian rhythm; interestingly, there was a small increase in plasma and salivary cortisol at 1800 h such that they were not different from the 0800 h result on day 1 or the 0600 h result on day 2. On average, the ESRD subjects did not achieve the full circadian nadir as the plasma cortisol was higher than controls between 1800 and 2400 h and the salivary cortisol was higher between 1200 and 2200 h. Plasma ACTH levels did not show a statistically detectable circadian rhythm in the ESRD subjects and were higher than controls from 1200 to 2400 h.

Using the conservative *a priori* criteria described in the Materials and methods section, the plasma and salivary cortisol levels were evaluated for the normality of the cortisol circadian rhythm. Twelve of the 16 ESRD subjects met the criteria for a normal circadian rhythm. Figure 1 (right panels) shows the plasma and salivary cortisol and plasma ACTH after removing from the analysis the data from the ESRD subjects not meeting the criteria of a normal cortisol rhythm. All the control subjects met the criteria for a normal rhythm. Even after removing four ESRD subjects that did not meet the criteria for a normal circadian rhythm from the analysis, the nighttime plasma and salivary cortisol levels were still significantly increased between 1600 and 2200 h compared with the control subjects.

**Figure 2:**
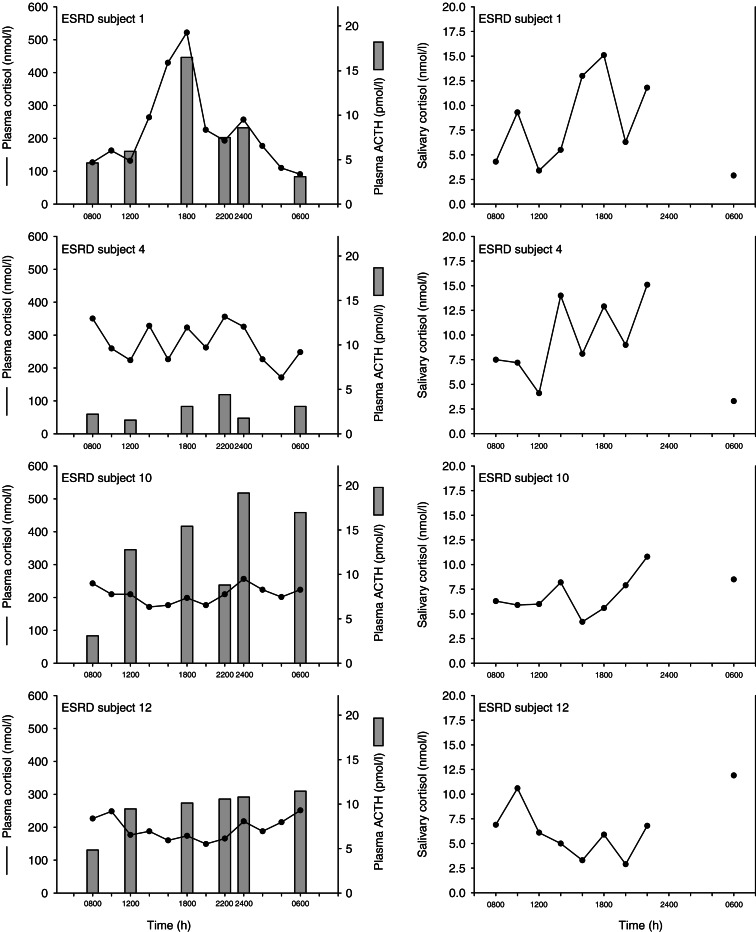
Plasma cortisol (lines) and ACTH (bars; left) and salivary cortisol (right) in the four subjects who did not meet the criteria for a normal cortisol circadian rhythm. Individual subjects are plotted from top to bottom.

Figure 2 shows the individual plasma and salivary cortisol and plasma ACTH data for the four ESRD subjects that did not meet the criteria for a normal circadian rhythm. Subject 1 seemed to have an inverted rhythm with a peak at 1800 h and a nadir at 0600 h. This subject was not a night shift worker, had daytime dialysis like the other ESRD subjects, and had a normal sleep diary for the night before and the night of sampling. The other three subjects had no discernable circadian rhythms and also had normal sleep diary records.

**Figure 3:**
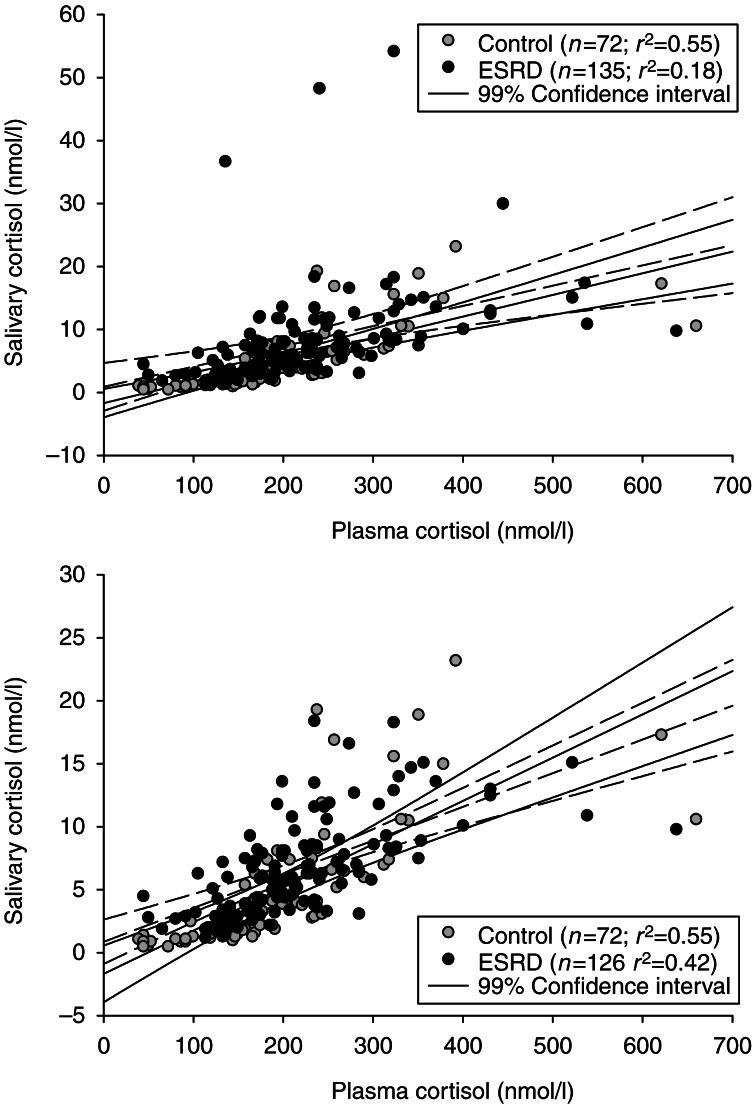
Correlation and linear regression (±99% confidence intervals) of individual plasma and salivary cortisol levels in all control vs ESRD subjects (top) and in all control vs ESRD subjects without the obviously contaminated saliva samples (bottom) (17). The slopes and intercepts were not different between control (solid line) and ESRD (dashed lined) subjects in either graph. For the top panel, control subjects slope and *y*-intercept was 0.034±0.004 and −1.7±0.9 nmol/l respectively and for ESRD subjects was 0.032±0.006 and 0.9±1.5 nmol/l respectively. For the bottom panel, the ESRD subjects slope and *y*-intercept was 0.027±0.003 and 0.9±0.7 nmol/l respectively. The Spearman's rank-order correlation coefficients were 0.902 for the controls (*P*<0.001) and 0.723 for the ESRD subjects (*P*<0.001).

Figure 3 shows the correlation of plasma and salivary cortisol individual results. First, note in the top panel that there were clearly contaminated salivary cortisol samples (increased salivary cortisol without increased plasma cortisol; see (17) for criteria for contamination of saliva samples). Even when this subject with contaminated saliva was eliminated from the analysis (bottom panel), there was no difference between the correlations of control vs ESRD subjects.

We were then interested in determining whether any of the baseline data of the ESRD subjects partitioned by circadian cortisol rhythm could offer any explanation for the four ESRD subjects with abnormal circadian rhythms shown in Fig. 2. As shown in Table 2, there were no statistically significant differences between the two ESRD groups partitioned by circadian rhythm. The only measurement that approached statistical difference was the duration on dialysis. Although formal sleep studies were not done simultaneously with cortisol sampling, analysis of the descriptive nurse's notes from the study did not identify any clear differences between the groups in Table 2. Serum calcium, phosphorus, and parathyroid hormone results were not different between the two ESRD groups (data not shown).

We used the small volume of remaining plasma (0800 h on day 1) from our subjects to measure CRP by a high-sensitivity assay to assess whether there could be association between a disrupted circadian HPA axis rhythm and an increased inflammatory state. Median (25–75% confidence intervals) plasma CRP levels (nmol/l) were 4.8 (3.9–59.0) in the control subjects, 53.5 (32.4–85.7) in the ESRD subjects with a normal cortisol circadian rhythm, and 147.6 (99.0–261.9) in the ESRD subjects with an abnormal circadian rhythm (one-way ANOVA on ranks; *P*=0.019).

## Discussion

The initial purpose of this study was to evaluate the usefulness of salivary cortisol to evaluate the HPA axis in subjects with ESRD. We have now clearly demonstrated that salivary cortisol is a useful surrogate for plasma cortisol in ESRD patients in agreement with many previous studies of patients with Cushing's syndrome, hypoalbuminemia, corticosteroid-binding globulin mutations, and adrenal insufficiency (6, 7, 18, 19).

We also found that ESRD subjects had a significantly increased nadir in salivary and plasma cortisol concentrations. When the four ESRD subjects with dramatically abnormal circadian rhythms were excluded from the analysis, the remaining ESRD subjects still had statistically significant increases in circadian nadir plasma and salivary cortisol levels. At first, we thought this could be due to changes in plasma proteins and/or cortisol metabolism and clearance. Arguing against this was the normal plasma albumin in ESRD subjects (Table 1), the excellent correlation of plasma and salivary cortisol (a validated surrogate for plasma free cortisol (1)), and the clearly abnormal plasma ACTH rhythm (Fig. 1). It should also be noted that the control subjects had a typical and normal circadian pattern of cortisol concentration indicating that the sampling conditions were appropriate.

It has been suggested that patients with ESRD have a decreased sensitivity to glucocorticoid-negative feedback assessed by dexamethasone suppression (10). That study did not take into account potential differences in dexamethasone absorption and metabolism nor did it evaluate physiological glucocorticoid-negative feedback sensitivity at different times of day. It also needs to be considered that direct immunoassays overestimate cortisol concentrations with dexamethasone suppression in ESRD patients (8, 20, 21). So it remains possible that ESRD patients have decreased glucocorticoid feedback sensitivity leading to an increase in plasma ACTH at the circadian nadir. One study demonstrated normal ACTH responses to ovine CRH in ESRD patients, whereas another from the same group suggested a blunted response indicating increased cortisol-negative feedback (22, 23). It is clear from our current study and previous studies that the relationship between plasma ACTH and cortisol appears to be intact in ESRD patients (24, 25).

Early studies showed normal cortisol rhythms in ESRD patients (10, 26), whereas more recent studies not specifically designed for this purpose have suggested a disrupted circadian rhythm (27, 28). The reason for this change over time is not clear, but it may be related to changes in characteristics of ESRD subjects. We do not think it is related to historical changes in the treatment of ESRD as there has been an improvement in the efficiency of hemodialysis therapy since the 1970s. Thus, if at all, one might expect metabolic abnormalities would be fewer and less severe in more recent years. The ESRD group tended to be younger than the control group. This age difference cannot account for the results we observed as older subjects, if anything, have increased late-night salivary cortisol and diminished sensitivity to glucocorticoid-negative feedback compared with younger subjects (29, 30, 31).

It was intriguing that small subset of ESRD subjects showed a dramatically abnormal ACTH and cortisol circadian rhythm. We could not identify any clinical correlate that could account for this (Table 2). The only variable that was even suggestive of a difference between ESRD subjects with normal and abnormal rhythms was the duration on dialysis, although there was clearly significant overlap of this variable between the two ESRD subgroups.

Subjects with chronic kidney disease have elevated markers of inflammation that are associated with a range of abnormalities including resistance to erythropoiesis-stimulating agents and increased morbidity (13). Although we did not initially intend to evaluate the possibility of a heightened inflammatory state, we were able to measure plasma CRP in the very small amount of plasma remaining at the end of the study. Our CRP data with admittedly small numbers of subjects are suggestive of an association of an increase in plasma CRP with a disrupted cortisol circadian rhythm. There is a well-documented increase in CRP in ESRD patients (32, 33) as well as an association of the immune and HPA axes (34, 35, 36). Although much work remains to be done on this topic and our CRP studies are *post hoc* and preliminary, it seems possible that ESRD patients with an increased inflammatory state may have a higher propensity for a disrupted circadian rhythm and/or decreased sensitivity to cortisol-negative feedback. Further, if these results are validated, further studies should investigate whether disrupted cortisol rhythm has clinical sequelae such as increased morbidity or mortality in ESRD subjects.

In conclusion, the measurement of salivary cortisol is a useful surrogate for plasma cortisol in patients with ESRD. This should facilitate the evaluation of these patients for the potential diagnosis of Cushing's syndrome. This is important as the stigmata of Cushing's syndrome are common in ESRD patients (13). In fact, it has been suggested that some of the sequelae of ESRD may be due to increased stress hormone concentrations (8, 9). It is also important to reiterate that salivary cortisol is a validated surrogate for plasma free cortisol – the biologically active component of the HPA axis. As we have described previously for elderly subjects, the exposure of the tissues and organs to twice or three times higher free cortisol concentration night after night for months to years is likely to result in significant negative consequences (29).

Taking into account the increased late-night cortisol levels, the increase in plasma ACTH, and previous studies showing diminished sensitivity to glucocorticoid feedback, we suggest that ESRD may represent a state of ACTH-dependent hypercortisolism. Therefore, from a diagnostic point of view, we propose that an increased late-night salivary cortisol in patients with ESRD should be interpreted with great caution. Whereas this measurement has a 95% sensitivity and specificity for Cushing's syndrome in patients without chronic kidney disease (6), we now suggest an increased likelihood of false-positive results in patients with ESRD, particularly considering the extremely low probability that any of the ESRD patients had endogenous Cushing's syndrome. This is similar to previous studies in patients with diabetes mellitus (37). It is important to reiterate that this study was powered to compare the circadian rhythms of salivary and plasma cortisol and that the findings of markedly abnormal salivary and plasma cortisol levels were somewhat of a surprise. An important next step would be to evaluate a large number of patients with various degrees of renal failure to establish disease-specific reference ranges for late-night salivary cortisol.

Therefore, if late-night salivary cortisol is normal, Cushing's syndrome can be ruled out in ESRD patients. However, we measured late-night salivary cortisol levels as high as 15 nmol/l in ESRD patients (clinical reference range <4.2 nmol/l). Therefore, because of both subtly and strikingly abnormal late-night plasma and salivary cortisol levels, the evaluation of ESRD patients for true Cushing's syndrome continues to be quite challenging.

## Figures and Tables

**Table 1:** Median (25–75% confidence intervals) baseline data on enrollment and baseline plasma data at 0800 h on the day of cortisol sampling.

**Subject** (*n*)	**Control** (8)	**ESRD** (16)
Age (years)	59 (49–63)	46 (35–56)*,a
Height (cm)^b^	178 (168–185)	174 (170–180)
Weight (kg)^b^	84 (75–88)	79 (68–88)
Male/female	6/2	14/2
Race (W, AA)	4/4	8/8
Serum creatinine (μmol/l)	80 (71–88)	
eGFR (ml/min)	94 (8–18)	
URR (%)		77 (71–79)
Duration on dialysis (months)		25 (9–56)
Serum albumin (g/l)		40 (38–43)
Serum bicarbonate (mmol/l)^c^		25.0 (23.3–26.6)

ESRD, end-stage renal disease; W, White; AA, African-American; eGFR, estimated glomerular filtration rate; URR, urea reduction ratio. Mann–Whitney rank sum test: *P<0.050.

a Age was not different by *t*-test (*P*=0.076).

b *n*=7 for control group.

c *n*=15 for ESRD group.

**Table 2:** Median (25–75% confidence intervals) baseline data on enrollment and baseline plasma data at 0800 h on the day of cortisol sampling for ESRD subjects partitioned by normality of cortisol circadian rhythm.

**Subject** (*n*)	**Normal rhythm** (12)	**Abnormal rhythm** (4)
Age (years)	46 (35–54)	52 (35–60)
Height (cm)	174 (169–180)	176 (171–181)
Weight (kg)^a^	83 (64–90)	74 (72–79)
Male/female	10/2	4/0
Race (W, AA)	5/7	1/3
Ethnicity (H/non-H)	1/11	0/4
URR (%)	79 (71–80)	76 (72–77)
Duration on dialysis (months)	25 (6–39)	82 (25–164)*
Dialysis access (*n*)		
AVG	2	1
AVF	9	2
PC	1	1
Serum albumin (g/l)	41 (38–44)	38 (34–40)
Serum bicarbonate (mmol/l)^b^	25.0 (23.3–26.6)	25.4 (22.0–26.4)

ESRD, end-stage renal disease; W, White; AA, African-American; H, Hispanic; eGFR, estimated glomerular filtration rate; URR, urea reduction ratio; AVG, arteriovenous graft; AVF, arteriovenous fistula; PC, Permcath. Mann–Whitney rank sum test: *P=0.162 (all other comparisons, *P*>0.200).

a *n*=3 for abnormal rhythm group.

b *n*=11 for normal rhythm group.
